# Supplementary material for: Identification of functional genetic components modulating toxicity response to PFOS using genome-wide CRISPR screens in HepG2/C3A cells
Source: Arch Toxicol. 2026 Jan 27;100(4):1391–410. doi: 10.1007/s00204-025-04294-8 (PMC13043545; doi:10.1007/s00204-025-04294-8)
Supplement: Supplementary file 3 — Supplementary Material 3 [file 204_2025_4294_MOESM3_ESM.docx]

**Primers for NGS preparation (LentiCRISPR V2 backbone)**

|  | **Primers** | **primer sequence** | **Index (8 b)** |
| --- | --- | --- | --- |
| Forward Primer | CRISPR-1step-For (F01) | AATGATACGGCGACCACCGAGATCTACACTCTTTCCCTACACGACGCTCTTCCGATCTNNNNNGCTTTATATATCTTGTGGAAAGGACGAAACACC | N/A |
| Reverse primers | CRISPR-1step-R01 | CAAGCAGAAGACGGCATACGAGATAAGTAGAGGTGACTGGAGTTCAGACGTGTGCTCTTCCGATCTCCGACTCGGTGCCACTTTTTCAA | AAGTAGAG |
| Reverse primers | CRISPR-1step-R02 | CAAGCAGAAGACGGCATACGAGATACACGATCGTGACTGGAGTTCAGACGTGTGCTCTTCCGATCTCCGACTCGGTGCCACTTTTTCAA | ACACGATC |
| Reverse primers | CRISPR-1step-R03 | CAAGCAGAAGACGGCATACGAGATCGCGCGGTGTGACTGGAGTTCAGACGTGTGCTCTTCCGATCTCCGACTCGGTGCCACTTTTTCAA | CGCGCGGT |
| Reverse primers | CRISPR-1step-R04 | CAAGCAGAAGACGGCATACGAGATCATGATCGGTGACTGGAGTTCAGACGTGTGCTCTTCCGATCTCCGACTCGGTGCCACTTTTTCAA | CATGATCG |
| Reverse primers | CRISPR-1step-R05 | CAAGCAGAAGACGGCATACGAGATCGTTACCAGTGACTGGAGTTCAGACGTGTGCTCTTCCGATCTCCGACTCGGTGCCACTTTTTCAA | CGTTACCA |
| Reverse primers | CRISPR-1step-R06 | CAAGCAGAAGACGGCATACGAGATTCCTTGGTGTGACTGGAGTTCAGACGTGTGCTCTTCCGATCTCCGACTCGGTGCCACTTTTTCAA | TCCTTGGT |
| Reverse primers | CRISPR-1step-R07 | CAAGCAGAAGACGGCATACGAGATAACGCATTGTGACTGGAGTTCAGACGTGTGCTCTTCCGATCTCCGACTCGGTGCCACTTTTTCAA | AACGCATT |
| Reverse primers | CRISPR-1step-R08 | CAAGCAGAAGACGGCATACGAGATACAGGTATGTGACTGGAGTTCAGACGTGTGCTCTTCCGATCTCCGACTCGGTGCCACTTTTTCAA | ACAGGTAT |
| Reverse primers | CRISPR-1step-R09 | CAAGCAGAAGACGGCATACGAGATAGGTAAGGGTGACTGGAGTTCAGACGTGTGCTCTTCCGATCTCCGACTCGGTGCCACTTTTTCAA | AGGTAAGG |
| Reverse primers | CRISPR-1step-R10 | CAAGCAGAAGACGGCATACGAGATAACAATGGGTGACTGGAGTTCAGACGTGTGCTCTTCCGATCTCCGACTCGGTGCCACTTTTTCAA | AACAATGG |
| Reverse primers | CRISPR-1step-R11 | CAAGCAGAAGACGGCATACGAGATACTGTATCGTGACTGGAGTTCAGACGTGTGCTCTTCCGATCTCCGACTCGGTGCCACTTTTTCAA | ACTGTATC |
| Reverse primers | CRISPR-1step-R12 | CAAGCAGAAGACGGCATACGAGATAGGTCGCAGTGACTGGAGTTCAGACGTGTGCTCTTCCGATCTCCGACTCGGTGCCACTTTTTCAA | AGGTCGCA |
| Reverse primers | CRISPR-1step-R13 | CAAGCAGAAGACGGCATACGAGATATAGGTCCGTGACTGGAGTTCAGACGTGTGCTCTTCCGATCTCCGACTCGGTGCCACTTTTTCAA | ATAGGTCC |
| Reverse primers | CRISPR-1step-R14 | CAAGCAGAAGACGGCATACGAGATCAGTGCTTGTGACTGGAGTTCAGACGTGTGCTCTTCCGATCTCCGACTCGGTGCCACTTTTTCAA | CAGTGCTT |
| Reverse primers | CRISPR-1step-R15 | CAAGCAGAAGACGGCATACGAGATACCATAGGGTGACTGGAGTTCAGACGTGTGCTCTTCCGATCTCCGACTCGGTGCCACTTTTTCAA | ACCATAGG |
| Reverse primers | CRISPR-1step-R16 | CAAGCAGAAGACGGCATACGAGATCAACTTGGGTGACTGGAGTTCAGACGTGTGCTCTTCCGATCTCCGACTCGGTGCCACTTTTTCAA | CAACTTGG |
| Reverse primers | CRISPR-1step-R17 | CAAGCAGAAGACGGCATACGAGATGACGAACTGTGACTGGAGTTCAGACGTGTGCTCTTCCGATCTCCGACTCGGTGCCACTTTTTCAA | GACGAACT |
| Reverse primers | CRISPR-1step-R18 | CAAGCAGAAGACGGCATACGAGATTCGATGACGTGACTGGAGTTCAGACGTGTGCTCTTCCGATCTCCGACTCGGTGCCACTTTTTCAA | TCGATGAC |
| Reverse primers | CRISPR-1step-R19 | CAAGCAGAAGACGGCATACGAGATCAAGTCGTGTGACTGGAGTTCAGACGTGTGCTCTTCCGATCTCCGACTCGGTGCCACTTTTTCAA | CAAGTCGT |
| Reverse primers | CRISPR-1step-R20 | CAAGCAGAAGACGGCATACGAGATAGTTCGCAGTGACTGGAGTTCAGACGTGTGCTCTTCCGATCTCCGACTCGGTGCCACTTTTTCAA | AGTTCGCA |
| Reverse primers | CRISPR-1step-R21 | CAAGCAGAAGACGGCATACGAGATCTGTATGCGTGACTGGAGTTCAGACGTGTGCTCTTCCGATCTCCGACTCGGTGCCACTTTTTCAA | CTGTATGC |
| Reverse primers | CRISPR-1step-R22 | CAAGCAGAAGACGGCATACGAGATTCTAGGAGGTGACTGGAGTTCAGACGTGTGCTCTTCCGATCTCCGACTCGGTGCCACTTTTTCAA | TCTAGGAG |
| Reverse primers | CRISPR-1step-R23 | CAAGCAGAAGACGGCATACGAGATTGCTTGCTGTGACTGGAGTTCAGACGTGTGCTCTTCCGATCTCCGACTCGGTGCCACTTTTTCAA | TGCTTGCT |
| Reverse primers | CRISPR-1step-R24 | CAAGCAGAAGACGGCATACGAGATACGAACGAGTGACTGGAGTTCAGACGTGTGCTCTTCCGATCTCCGACTCGGTGCCACTTTTTCAA | ACGAACGA |
| Reverse primers | CRISPR-1step-R25 | CAAGCAGAAGACGGCATACGAGATTTCGCCATGTGACTGGAGTTCAGACGTGTGCTCTTCCGATCTCCGACTCGGTGCCACTTTTTCAA | TTCGCCAT |
| Reverse primers | CRISPR-1step-R26 | CAAGCAGAAGACGGCATACGAGATGAGCAATCGTGACTGGAGTTCAGACGTGTGCTCTTCCGATCTCCGACTCGGTGCCACTTTTTCAA | GAGCAATC |
| Reverse primers | CRISPR-1step-R27 | CAAGCAGAAGACGGCATACGAGATAACACTGGGTGACTGGAGTTCAGACGTGTGCTCTTCCGATCTCCGACTCGGTGCCACTTTTTCAA | AACACTGG |
| Reverse primers | CRISPR-1step-R28 | CAAGCAGAAGACGGCATACGAGATCCATGAACGTGACTGGAGTTCAGACGTGTGCTCTTCCGATCTCCGACTCGGTGCCACTTTTTCAA | CCATGAAC |
| Reverse primers | CRISPR-1step-R29 | CAAGCAGAAGACGGCATACGAGATATAGTCGGGTGACTGGAGTTCAGACGTGTGCTCTTCCGATCTCCGACTCGGTGCCACTTTTTCAA | ATAGTCGG |
| Reverse primers | CRISPR-1step-R30 | CAAGCAGAAGACGGCATACGAGATGATTGTCCGTGACTGGAGTTCAGACGTGTGCTCTTCCGATCTCCGACTCGGTGCCACTTTTTCAA | GATTGTCC |
| Reverse primers | CRISPR-1step-R31 | CAAGCAGAAGACGGCATACGAGATTCCACGTTGTGACTGGAGTTCAGACGTGTGCTCTTCCGATCTCCGACTCGGTGCCACTTTTTCAA | TCCACGTT |
| Reverse primers | CRISPR-1step-R32 | CAAGCAGAAGACGGCATACGAGATCAACTCCAGTGACTGGAGTTCAGACGTGTGCTCTTCCGATCTCCGACTCGGTGCCACTTTTTCAA | CAACTCCA |
| Reverse primers | CRISPR-1step-R33 | CAAGCAGAAGACGGCATACGAGATTGGTGAAGGTGACTGGAGTTCAGACGTGTGCTCTTCCGATCTCCGACTCGGTGCCACTTTTTCAA | TGGTGAAG |
| Reverse primers | CRISPR-1step-R34 | CAAGCAGAAGACGGCATACGAGATTGAGCTGTGTGACTGGAGTTCAGACGTGTGCTCTTCCGATCTCCGACTCGGTGCCACTTTTTCAA | TGAGCTGT |
| Reverse primers | CRISPR-1step-R35 | CAAGCAGAAGACGGCATACGAGATAACCAGAGGTGACTGGAGTTCAGACGTGTGCTCTTCCGATCTCCGACTCGGTGCCACTTTTTCAA | AACCAGAG |
| Reverse primers | CRISPR-1step-R36 | CAAGCAGAAGACGGCATACGAGATAAGTCCTCGTGACTGGAGTTCAGACGTGTGCTCTTCCGATCTCCGACTCGGTGCCACTTTTTCAA | AAGTCCTC |
| Reverse primers | CRISPR-1step-R37 | CAAGCAGAAGACGGCATACGAGATGTTCTTCGGTGACTGGAGTTCAGACGTGTGCTCTTCCGATCTCCGACTCGGTGCCACTTTTTCAA | GTTCTTCG |
| Reverse primers | CRISPR-1step-R38 | CAAGCAGAAGACGGCATACGAGATACAGTGACGTGACTGGAGTTCAGACGTGTGCTCTTCCGATCTCCGACTCGGTGCCACTTTTTCAA | ACAGTGAC |
| Reverse primers | CRISPR-1step-R39 | CAAGCAGAAGACGGCATACGAGATCGCAATGTGTGACTGGAGTTCAGACGTGTGCTCTTCCGATCTCCGACTCGGTGCCACTTTTTCAA | CGCAATGT |
| Reverse primers | CRISPR-1step-R40 | CAAGCAGAAGACGGCATACGAGATAACCGTGTGTGACTGGAGTTCAGACGTGTGCTCTTCCGATCTCCGACTCGGTGCCACTTTTTCAA | AACCGTGT |
| Reverse primers | CRISPR-1step-R41 | CAAGCAGAAGACGGCATACGAGATCCGTTATGGTGACTGGAGTTCAGACGTGTGCTCTTCCGATCTCCGACTCGGTGCCACTTTTTCAA | CCGTTATG |
| Reverse primers | CRISPR-1step-R42 | CAAGCAGAAGACGGCATACGAGATCCACAACAGTGACTGGAGTTCAGACGTGTGCTCTTCCGATCTCCGACTCGGTGCCACTTTTTCAA | CCACAACA |
| Reverse primers | CRISPR-1step-R43 | CAAGCAGAAGACGGCATACGAGATGGTACGAAGTGACTGGAGTTCAGACGTGTGCTCTTCCGATCTCCGACTCGGTGCCACTTTTTCAA | GGTACGAA |
| Reverse primers | CRISPR-1step-R44 | CAAGCAGAAGACGGCATACGAGATTACTGCTCGTGACTGGAGTTCAGACGTGTGCTCTTCCGATCTCCGACTCGGTGCCACTTTTTCAA | TACTGCTC |
| Reverse primers | CRISPR-1step-R45 | CAAGCAGAAGACGGCATACGAGATACCTCTTCGTGACTGGAGTTCAGACGTGTGCTCTTCCGATCTCCGACTCGGTGCCACTTTTTCAA | ACCTCTTC |
| Reverse primers | CRISPR-1step-R46 | CAAGCAGAAGACGGCATACGAGATTGGATGGTGTGACTGGAGTTCAGACGTGTGCTCTTCCGATCTCCGACTCGGTGCCACTTTTTCAA | TGGATGGT |
| Reverse primers | CRISPR-1step-R47 | CAAGCAGAAGACGGCATACGAGATCTTCACTGGTGACTGGAGTTCAGACGTGTGCTCTTCCGATCTCCGACTCGGTGCCACTTTTTCAA | CTTCACTG |
| Reverse primers | CRISPR-1step-R48 | CAAGCAGAAGACGGCATACGAGATTACTAGCGGTGACTGGAGTTCAGACGTGTGCTCTTCCGATCTCCGACTCGGTGCCACTTTTTCAA | TACTAGCG |
